# Novel application of one-step pooled molecular testing and maximum likelihood approaches to estimate the prevalence of malaria parasitaemia among rapid diagnostic test negative samples in western Kenya

**DOI:** 10.1186/s12936-022-04323-2

**Published:** 2022-11-06

**Authors:** Monica P. Shah, Winnie Chebore, Robert H. Lyles, Kephas Otieno, Zhiyong Zhou, Mateusz Plucinski, Lance A. Waller, Wycliffe Odongo, Kim A. Lindblade, Simon Kariuki, Aaron M. Samuels, Meghna Desai, Rebecca M. Mitchell, Ya Ping Shi

**Affiliations:** 1 Division of Parasitic Diseases and Malaria, Center for Global Health, Centers for Disease Control and Prevention, Atlanta, GA USA; 2 Department of Epidemiology, Rollins School of Public Health, Emory University, Atlanta, GA USA; 3 Kenya Medical Research Institute, Centre for Global Health Research, Kisumu, Kenya; 4 Department of Biostatistics and Bioinformatics, Rollins School of Public Health, Emory University, Atlanta, GA USA; 5 Department of Computer Science, Emory University, Atlanta, GA USA

**Keywords:** Pooled testing, Group testing, Subpatent malaria parasitemia

## Abstract

**Background:**

Detection of malaria parasitaemia in samples that are negative by rapid diagnostic tests (RDTs) requires resource-intensive molecular tools. While pooled testing using a two-step strategy provides a cost-saving alternative to the gold standard of individual sample testing, statistical adjustments are needed to improve accuracy of prevalence estimates for a single step pooled testing strategy.

**Methods:**

A random sample of 4670 malaria RDT negative dried blood spot samples were selected from a mass testing and treatment trial in Asembo, Gem, and Karemo, western Kenya. Samples were tested for malaria individually and in pools of five, 934 pools, by one-step quantitative polymerase chain reaction (qPCR). Maximum likelihood approaches were used to estimate subpatent parasitaemia (RDT-negative, qPCR-positive) prevalence by pooling, assuming poolwise sensitivity and specificity was either 100% (strategy A) or imperfect (strategy B). To improve and illustrate the practicality of this estimation approach, a validation study was constructed from pools allocated at random into main (734 pools) and validation (200 pools) subsets. Prevalence was estimated using strategies A and B and an inverse-variance weighted estimator and estimates were weighted to account for differential sampling rates by area.

**Results:**

The prevalence of subpatent parasitaemia was 14.5% (95% CI 13.6–15.3%) by individual qPCR, 9.5% (95% CI (8.5–10.5%) by strategy A, and 13.9% (95% CI 12.6–15.2%) by strategy B. In the validation study, the prevalence by individual qPCR was 13.5% (95% CI 12.4–14.7%) in the main subset, 8.9% (95% CI 7.9–9.9%) by strategy A, 11.4% (95% CI 9.9–12.9%) by strategy B, and 12.8% (95% CI 11.2–14.3%) using inverse-variance weighted estimator from poolwise validation. Pooling, including a 20% validation subset, reduced costs by 52% compared to individual testing.

**Conclusions:**

Compared to individual testing, a one-step pooled testing strategy with an internal validation subset can provide accurate prevalence estimates of PCR-positivity among RDT-negatives at a lower cost.

**Supplementary Information:**

The online version contains supplementary material available at 10.1186/s12936-022-04323-2.

## Background

Subpatent malaria infections are defined as those with parasite densities below thresholds detectable by routine tests such as conventional malaria rapid diagnostic tests (RDT) and microscopy. In high transmission settings, subpatent infections may account for up to 20% of all malaria infections [1] and are typically asymptomatic. Subpatent infections contribute to the human infectious parasite reservoir as a proportion of these infections can infect mosquitos and contribute to transmission [2, 3]. In the absence of care-seeking behaviour prompted by symptoms, subpatent infections can persist for weeks to months [4, 5]. However, detection of subpatent infections requires resource-intensive molecular tests with very low detection limits, such as polymerase chain reaction (PCR) [6], which limits large-scale estimation of population prevalence and routine surveillance efforts.

For less prevalent conditions, pooled (or group) testing strategies have been proposed as an efficient and cost-effective alternative to individual sample testing for either large-scale screening for disease or estimating overall prevalence of disease [7]. Several frequently cited applications include the use of pooling for screening of blood donors for HIV antibodies [8], screening for syphilis [9], disease prevalence estimation in veterinary medicine [10], and, more recently, optimizing testing capacity for COVID-19 [11]. Optimal pool sizes are commonly determined based on the expected prevalence of the disease and characteristics of the diagnostic test. For estimates of disease prevalence, samples should be allocated at random to a pool.

Pooled testing designs can include single- or multiple-steps with a fixed or varied pool size [12]. For instance, in a two-step pooled approach, all pools are tested in the first round and individual samples from positive pools undergo a second round of testing. Maximum likelihood estimation (MLE) or Bayesian methods can be used to estimate the prevalence and precision from pooled tests either assuming perfect (100%) or accounting for imperfect sensitivity and specificity [13].

Pooled testing has been used in low malaria transmission settings to estimate malaria prevalence among pregnant women [14, 15] and children [16], to identify drug-resistant molecular markers in pregnant women [17], and to detect malaria in active case detection strategies [18, 19]. In high transmission settings, estimation of population-level malaria prevalence using pooled testing would be inefficient; low parasite density infections, which are often missed by routine diagnostic tests, make up a relatively smaller proportion of all malaria infections overall in high transmission settings. Therefore, when combined with a routine test negative sampling approach, pooled testing may provide a cost-effective alternative to individual sampling testing for the estimation of malaria parasitaemia prevalence among RDT- or microscopy-negative samples.

The present study had four objectives: (1) to estimate the prevalence of malaria parasitaemia among RDT negative samples (defined as a RDT negative/PCR positive infection, irrespective of parasite density) using a one-step pooled testing strategy and a novel application of MLE, (2) to describe the validity of the pooled approach relative to individual PCR testing and characterize trends in sensitivity, (3) to conduct a validation study to illustrate methods to improve estimates in future pooled testing studies, and (4) to estimate the resource savings of the pooled testing approach.

## Methods

### Ethical considerations

The study protocol was approved by the Kenya Medical Research Institute (KEMRI) institutional review board (IRB); the U.S. Centers for Disease Control and Prevention (CDC) relied on KEMRI for approval. The original trial was registered at ClinicalTrials.gov (NCT02987270). Written informed consent including sample collection and molecular testing was obtained from adult participants and parents/guardians of participating children and written informed assent was sought for adolescent participants 13–17 years of age.

### Study population and area

Samples for this study were collected from individuals enrolled in a mass test and treat (MTaT) trial conducted in the high transmission areas of Asembo, Gem, and Karemo in Siaya County, western Kenya between 2013–2015. At each round, all individuals in intervention clusters were tested for malaria by RDT (Carestart™ Malaria HRP-2/pLDH [Pf/PAN] Combo Test RDT; Somerset, NJ, USA), those that were positive were treated with an anti-malarial, and a dried blood spot (DBS) was collected on filter paper (Whatman 903 Proteinsaver Card, Whatman Inc, Piscataway, NJ) [20–23].

### Sample size and selection

The present study used DBSs from a random sample collected from RDT-negative participants during the first round of MTaT in October 2013. Sampling was stratified by area based on Round 1 RDT prevalence as a proxy for transmission intensity: Asembo (lowest relative to study area, RDT prevalence 36%), Gem (39%), and Karemo (highest, 51%) (unpublished data). A pilot exercise was conducted to determine the sample and pool size for this study (Additional file 1). A stratified random sample of 4,978 DBSs (1,925 from Asembo, 2,228 from Gem, and 825 from Karemo) was selected for molecular analysis by qPCR. Among these, 308 (6.2%) samples were excluded due to inability to be located (DBS collected, but not found in the laboratory), insufficient blood volume on DBS, or dropped to allocate pool sizes of 5 samples within transmission strata, resulting in 4,670 samples (934 pools) analysed. Within each transmission area, samples were assigned at random to a pool.

### Laboratory methods: DNA extraction and qPCR amplification

Following collection, DBS samples were dried overnight at room temperature and sealed individually with desiccant and a moisture indicator the next day. Samples were stored at − 80 °C until DNA extraction.

All procedures were carried out in parallel for pooled and individual assays to minimize variability. For pooled tests, a single 5 mm circular paper disk (equivalent to 50 μl of blood) was cut from each selected DBS and five disks were combined for DNA extraction. Simultaneously, a second circular disk was cut for individual DNA extraction. DNA was extracted according to the manufacturer’s protocol specified in QIAamp DNA Mini Kits (QIAGEN, Valencia, CA) with the following exceptions for pooled samples: doubled volume of lysis buffer and an additional cleaning step using OneStep PCR inhibitor removal kit (Zymo Research Corp, Irvine, CA). The elution volume for both pooled and individual samples was 50 μl.

Samples were tested both individually and in pool sizes of five on the same plate by qPCR amplification of the *Plasmodium falciparum* 18S small subunit ribosome RNA gene qPCR [24]. Reactions of 20 μl were prepared with 2.5 μl of DNA template (same volume for pooled and individual samples) in 1 × TaqMan® Universal qPCR Master Mix (Applied Biosystems, Foster City, CA), 300 nM of each primer (Forward: 5’ GTA ATT GGA ATG ATA GGA ATT TAC AAG GT 3’; Reverse: 5’ TCA ACT ACG AAC GTT TTA ACT GCA AC 3’) and 150 nM of TaqMan probe labelled with 6-carboxy-fluorescein (FAM) as a reporter and 6-carboxytetramethylrhodamine (TAMRA) as a quencher (5’-FAM-TGC CAG CAG CCG CGG TAA TTC-TAMRA). All reactions were performed on a Stratagene Mx3005P qPCR system with the following steps: initial denaturation at 95 °C for 10 min, 40 cycles at 95 °C for 15 s, and 60 °C for 1 min. Each plate included five 10 × standard curve dilutions from 5 to 5 × 10^4^ parasites per μl and negative control wells run in triplicate. Field samples were run in duplicate.

Samples that did not amplify (no cycle threshold, C_t_, value detected) were considered negative. Parasite density (parasites per µL) of field samples was determined based on the known standard curve concentrations and values between 0 and the minimum standard curve dilution of 5 were recorded as 2 parasites per µL.

### Data and statistical analyses

#### Sensitivity, specificity, and prevalence estimation.

The sensitivity (se) and specificity (sp) of one-step pooled qPCR testing were defined as:

1 $$Se= P\left(Pool\,test+ \right|\,at\,least\,one\,individual\,sample\,in\,pool\,was\,test +)$$

2 $$Sp= P\left(Pool\,test- \right|\,all\,individual\,samples\,in\,pool\,were\,test -)$$

The prevalence of subpatent parasitaemia and corresponding 95% confidence intervals (CI) for individual qPCR and one-step pooled qPCR tests were estimated using MLE procedures summarized in Cowling et al. [13] and derived from Kline et al. [8] and Tu et al. [25]. For pooled tests, the individual-level prevalence (π) estimate and corresponding standard error (σ) given *x* positive pools, *m* pools tested, a pool size of *k* (five), and MLE of pool-level prevalence $\widehat{P}=x/m$ were initially derived assuming perfect sensitivity and specificity (*se* = *sp* = 100%; see Eqs. 3a and 3b). Subsequently, π and σ were estimated adjusting for imperfect sensitivity and specificity (see Eqs. 4a and 4b), with Se and Sp estimated from the data according to Eqs. 1 and 2. Analyses were conducted by area and by all areas combined, after weighting area-specific prevalence estimates by population (Additional file 1). The variation in sensitivity of one-step pooled qPCR was also explored as a function of the number of true positive samples in the pool. Additionally, we examined trends in prevalence, sensitivity, and specificity by area to explore variation by transmission settings.

3a $${\widehat{\pi }}_{MLE}={1-\left(1-\widehat{P}\right)}^{1/k}$$

3b $${\widehat{\sigma }}^{2}({\widehat{\pi }}_{MLE})=\frac{{\widehat{P}\left(1-\widehat{P}\right)}^{\frac{2}{k}-1}}{m{k}^{2}}$$

4a $${{\widehat{\pi }}^{*}}_{MLE}={1-\left(\frac{Se-\widehat{P}}{Se+Sp-1}\right)}^{1/k}$$

4b $${\widehat{\sigma }}^{2}({{\widehat{\pi }}^{*}}_{MLE})=\frac{{(1-{{\widehat{\pi }}^{*}}_{MLE})}^{2-2k}}{m{k}^{2}}\frac{\widehat{P}\left(1-\widehat{P}\right)}{{(Se+Sp-1)}^{2}}$$

### Validation study to improve prevalence estimators

Since a main advantage of pooled testing is to minimize the need for individual tests, we conducted a validation study to demonstrate methods to estimate sensitivity and specificity in a validation subset and used these results to correct prevalence estimators and account for variability in sensitivity and specificity estimates in the variance of the prevalence estimate.

A validation subset was constructed by selecting a simple random sample of 200 pools (n = 1000 individual samples) or approximately 21% of the whole dataset. Sensitivity and specificity of one-step pooled testing were estimated based on Eqs. 1 and 2. Within the remaining 734 pools (n = 3670 samples), designated as the main study subset, we estimated prevalence and the corresponding standard error using four approaches: 1) MLE assuming perfect sensitivity and specificity (Eqs. 3a and 3b), 2) MLE adjusting for imperfect sensitivity and specificity, treating estimated Se and Sp obtained in the validation subset as if they were known to be correct (Eqs. 4a and 4b), 3) MLE adjusting for imperfect sensitivity and specificity (same prevalence estimate as in #2), with standard error based on a delta method approximation [26] to account for sampling variability with the Se and Sp estimates treated as if from an external validation sample, and 4) an inverse-variance weighted prevalence estimator and standard error appropriately incorporating the combined main and validation data and accounting for the internal nature of the validation subset [26–28]. As described in Thomas et al. [29], validation samples derived from outside the main study are treated as external and those derived as a subset of the main study are considered internal. Since the purpose of the validation subset was to provide an illustrative example of this methodology, overall prevalence estimates were not weighted by area.

## Parasite density

The average parasite density as a continuous measure and the proportion of low-density infections (defined as < 100 parasites per µL) among positive individual PCR tests were described overall and by transmission area. Among pools with at least one sample positive by individual PCR, the average parasite density was estimated and compared for true positive and false negative pools. Parasite density was log transformed prior to any statistical testing.

## Cost- and time-savings

Using the number of samples and pools run in this analysis, the cost savings of pooled compared to individual qPCR tests was determined based on consumable costs including DNA extraction kits and reagents, qPCR mastermix, probes, primers, and PCR plates, and general supplies including tubes, pipette tips, and gloves. Time requirements were estimated based on hands-on staff hours needed to cut DBSs, extract DNA, and prepare samples for qPCR.

For statistical inference, Chi-square tests were used to compare proportions and analysis of variance (ANOVA) to compare population means. P-values < 0.05 were considered statistically significant. Data management was carried out in SAS v9.4 (SAS Institute, Cary, North Carolina) and all analyses were conducted using RStudio v1.1.453 (RStudio Team, 2018) and R 3.6.1 (R Core Team, 2019).

## Results

### Sensitivity and specificity of one-step pooled qPCR

Given at least one sample tested positive by individual PCR in the positive pool, the sensitivity of one-step pooled qPCR was 74.5% (95% CI 70.5–78.5%) and, given all five samples tested negative by individual qPCR in the negative pool, the specificity was 98.3% (95% CI 97.1–99.5%). The sensitivity was inversely related to transmission setting, with the highest sensitivity in the lowest transmission area of Asembo (79.2%, 95% CI 72.5–85.8%), followed by Gem (73.7%, 95% CI 67.9–79.6%), and then Karemo (69.6%, 95% CI 60.7–78.5%). Specificity also showed a similar trend, but the decline was much smaller: 99.0% (95% CI 97.7–100.0%) in Asembo, 98.1% (95% CI 96.3–99.9%) in Gem, and 96.4% (95% CI 91.6–100.0%) in Karemo (Table 1).

**Table 1:** Estimates of malaria parasitemia prevalence among rapid diagnostic test negative samples by individual and one-step pooled testing by quantitative polymerase chain reaction

Study area	qPCR testing strategy	Prevalence estimation method	Samples or pools tested, N	Positive, n	Pool size	Sensitivity, % (95% CI)	Specificity, % (95% CI)	Prevalence, % (95% CI)
Asembo	Individual	Binomial	1735	189	1	ref.	ref.	10.9 (9.5–12.5)
	One-step pooled	MLE, se = sp = 1	347	116	5	79.2 (72.5–85.8)	99.0 (97.7–100.0)	7.8 (6.4–9.2)
	One-step pooled	MLE, se = 0.792, sp = 0.990	347	116	5			10.2 (8.2–12.1)^a^
Gem	Individual	Binomial	2145	303	1	ref.	ref.	14.1 (12.7–15.7)
	One-step pooled	MLE, se = sp = 1	429	164	5	73.7 (67.9–79.6)	98.1 (96.3–99.9)	9.2 (7.8–10.5)
	One-step pooled	MLE, se = 0.737, sp = 0.981	429	164	5			13.2 (10.9–15.5)^a^
Karemo	Individual	Binomial	790	145	1	ref.	ref.	18.4 (15.8–21.2)
	One-step pooled	MLE, se = sp = 1	158	73	5	69.6 (60.7–78.5)	96.4 (91.6–100.0)	11.7 (9.1–14.2)
	One-step pooled	MLE, se = 0.696, sp = 0.964	158	73	5			18.7 (13.3–24.1)^a^
Overall	Individual	Binomial	4670	637	1	ref.	ref.	14.5 (13.6–15.3) ^b^
	One-step pooled	MLE, se = sp = 1	934	353	5	74.5 (70.5–78.5)	98.3 (97.1–99.5)	9.5 (8.5–10.5) ^b^
	One-step pooled	MLE, se = 0.745, sp = 0.983	934	353	5			13.9 (12.6–15.2) ^a,b^

*CI*  confidence interval, *MLE*  maximum likelihood estimation, *qPCR*  quantitative polymerase chain reaction, *ref* = reference, *Se*  sensitivity, *Sp*  specificity

^a^Assumes fixed estimates of sensitivity and specificity denoted under prevalence estimation method

^b^Weights were applied to overall estimates to account for differential sampling rates by area

**Fig. 1:**
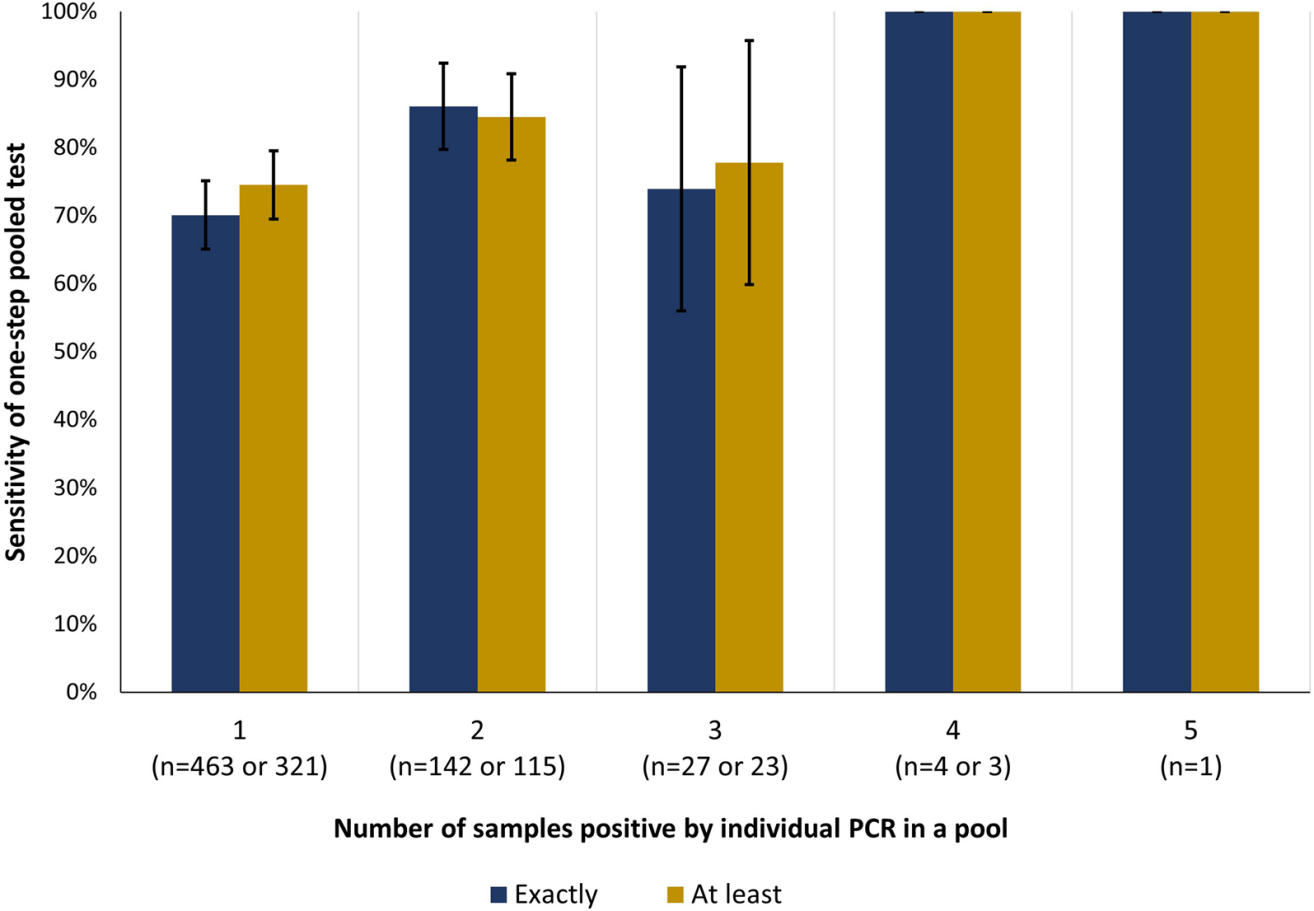
Sensitivity of one-step pooled quantitative polymerase chain reaction test given exactly or at least *n* true positive samples in a pool. Error bars indicate 95% confidence interval around sensitivity estimate

Additionally, as the number of positive samples in a pool increased (based on individual qPCR result), the probability of a positive pool result generally also increased, although not linearly. The trend was similar when viewed as a function of exactly *n* positive samples, or as a function of at least *n* positive samples in a pool. While sensitivity was 100% for 4 and 5 true positive samples, only 4 pools had 4 or more true positive samples (Fig. 1).

### Estimation of subpatent parasitaemia prevalence

Across all areas, the prevalence of subpatent (RDT negative, qPCR positive) parasitaemia by individual qPCR testing was 14.5% (95% CI 13.6–15.3%, gold standard prevalence). One-step pooled qPCR assuming 100% sensitivity and specificity underestimated the gold standard prevalence (9.5%, 95% CI 8.5–10.5%). After correcting for the poolwise sensitivity and specificity generated from our study data (described in the next section), the one-step pooled qPCR prevalence estimate improved to 13.9% (95% CI 12.6–15.2%) (Table 1).

Estimates of subpatent parasitaemia differed by transmission setting. In Asembo, Gem, and Karemo, respectively, the prevalence by individual qPCR was 10.9%, 14.1%, and 18.4%. By one-step pooled PCR, the prevalence was 7.8%, 9.2%, and 11.7%, respectively. After adjustment for the sensitivity and specificity of the pooled test, the prevalence was estimated as 10.2% in Asembo, 13.2% in Gem, and 18.7% in Karemo (Table 1).

### Validation study

Within the randomly selected validation subset (n = 200 pools, n = 1000 samples), the prevalence of malaria parasitaemia among RDT negatives by individual qPCR testing was 14.1% (95% CI 12.1–16.4%) and the estimated sensitivity and specificity of one-step pooled testing were 79.6% (95% CI 71.6–87.6%) and 98.0% (95% CI 95.3–100.0%), respectively (Table 2).

**Table 2:** Validation study of prevalence estimation approaches and accounting for variability in sensitivity and specificity of one-step pooled testing

qPCR testing strategy	Prevalence estimation method	Samples or pools tested, N	Positive, n	Pool size	Prevalence, % (95% CI)
Individual	Binomial (validation subset)^a^	1000	141	1	14.1 (12.1–16.4)
Individual	Binomial (main subset)^b^	3670	496	1	13.5 (12.4–14.7)
One-step pooled	MLE, se = sp = 1	734	273	5	8.9 (7.9–9.9)
One-step pooled	MLE, se = 0.796^a^, sp = 0.980^a^	734	273	5	11.4 (9.9–12.9)
One-step pooled	MLE, se = 0.796^a^, sp = 0.980^a^, accounting for σ^2^_SE_ and σ^2^_SP_	734	273	5	11.4 (9.2–13.6)
One-step pooled	Inverse-weighted prevalence estimator accounting for σ^2^_SE_ and σ^2^_SP_	734	273	5	12.8 (11.2–14.3)

*CI*  confidence interval, *MLE*  maximum likelihood estimation, *qPCR*  quantitative polymerase chain reaction, *Se*  sensitivity, *Sp* specificity, σ^2^_SE_  variance in sensitivity, σ^2^_SP_  variance in specificity

^a^Estimated from validation subset (n = 200 pools, n = 1000 samples); se = 0.796 (95% CI: 0.716–0.876), sp = 0.980 (95%CI: 0.953–1.000)

^b^Typically unknown in a study, but it is included here to illustrate the gold standard estimate

For illustrative purposes, the gold standard prevalence of subpatent parasitaemia by individual qPCR testing in the main study subset (n = 3670) was 13.5% (95% CI 12.4–14.7%). The accuracy and precision of estimates provided by pooled testing varied by estimation approach. Assuming 100% sensitivity and specificity, the prevalence of subpatent parasitaemia by one-step pooled qPCR was 8.9% (95% CI 7.9–9.9%). Accounting for imperfect sensitivity and specificity determined from the validation subset (and treating that subset as external to the main study) increased the estimate to 11.4% (95% CI 9.9–12.9) and the precision of this estimate widened the 95% CI to (9.2–13.6%) after incorporating the variance of the sensitivity and specificity estimate using a delta method approximation. Combining both the validation subset (appropriately treated as internal) and the main study data and applying inverse variance weighting provided a prevalence estimate of 12.8% (95% CI 11.2–14.3%), closest to the gold standard prevalence (Table 2).

### Parasitaemia density

**Fig. 2:**
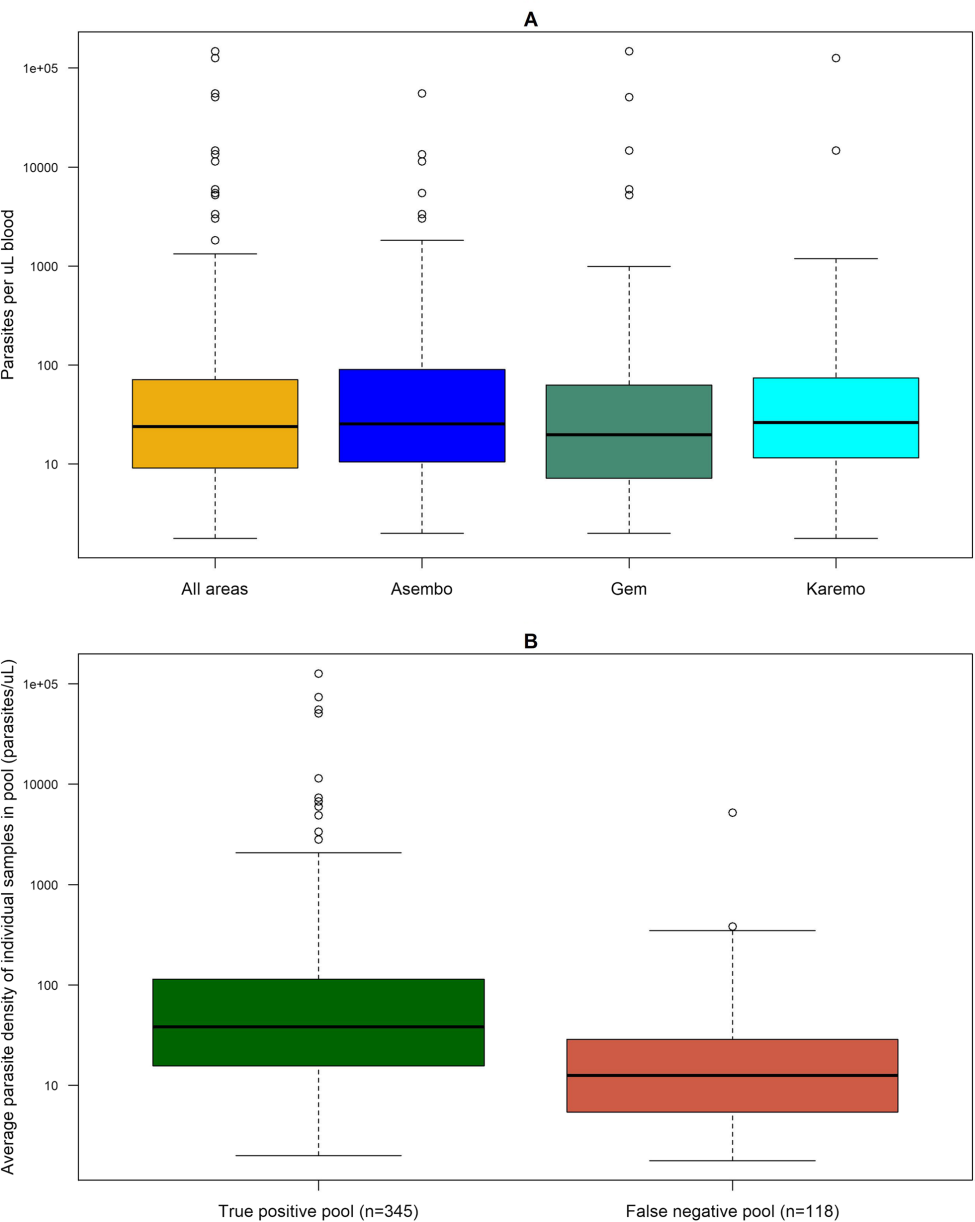
Parasite density among samples positive by individual quantitative polymerase chain reaction by **A** transmission area and **B** pooled testing result

Among samples positive by individual qPCR (n = 637), the geometric mean parasite density (GMPD) was 29.0 parasites per µL (median 23.9, interquartile range [IQR]: 9.1–71.5). There was a significant difference in GMPD across study areas, but this did not correspond with transmission intensity (one-way ANOVA p-value = 0.0317) (Fig. 2A). Overall, 80.1% of qPCR positive infections were classified as low density parasitaemia (< 100 parasites per µL) and this proportion increased with transmission intensity (8.3% in Asembo, 11.7% in Gem, and 14.6% in Karemo, chi-square test p-value < 0.0001).

There was at least one positive individual sample in 463 pools, of which 345 (74.5%) were positive by pooled qPCR (true positive). The average parasite density among positive individual samples in a given pool was significantly higher in true positive pools (GMPD 48.6, median 38.3, IQR 15.6–114.4) compared to false negative pools (GMPD 14.2, median 12.5, IQR 5.5–28.5, t-test p-value < 0.0001) (Fig. 2B).

### Cost and time comparisons of individual versus one-step pooled tests

The reagent and supply costs of running 4,670 samples individually was USD $22,509 compared to $6,090 for one-step pooled qPCR of 934 pools of 5 samples, which resulted in a cost savings of 73%. The estimated personnel hands-on time required to run one-step pooled qPCR, including the most labor-intensive step of DNA extraction, was 42% lower than individual qPCR tests (448 h versus 771 h). Inclusion of a validation subset of 1000 samples (200 pools for validation, plus 734 remaining pools) still resulted in cost (52% reduction) and time (20% reduction) savings compared to individual qPCR testing (Table 3).

**Table 3:** Comparison of resource costs and time required for individual and one-step pooled quantitative polymerase chain reaction tests

Test	Number of tests	Unit	Total cost, USD^a^	Total time, hours^b^	Total cost savings, USD	Total time savings, hours
Individual qPCR	4670	samples	$22,509^c^	771^d^	ref.	ref.
One-step pooled qPCR	934	pools of 5	$6,090^e^	448^f^	$16,420 (73%)	322 (42%)
One-step pooled qPCR with validation subset	934 pools and 1000 samples		$10,910	613	$11,600 (52%)	157 (20%)

qPCR = quantitative polymerase chain reaction, ref = reference, USD = United States Dollar

^a^Costs include: DNA extraction kits and reagents, cleaning reagents for pooled sample extraction, qPCR mastermix, probes, primers, and qPCR plates, and general supplies including tubes, pipette tips, and gloves, etc.

^b^Includes hands-on staff time required to cut dried blood spots, extract DNA, and prepare samples for qPCR

^c^Estimated cost per test: USD $4.82

^d^Estimated time for 1000 samples or 200 pools: 165 h

^e^Estimated cost per test: USD $6.52

^f^Estimated time for 1000 samples or 200 pools: 96 h

## Discussion

This study describes a novel application of one-step pooled testing strategies and MLE approaches in a high malaria transmission setting to estimate the prevalence of malaria parasitaemia in RDT negative samples. The results show that a one-step pooled molecular test can provide accurate estimates of prevalence after adjusting for sensitivity and specificity in comparison to individual sample qPCR testing. This study also shows that including information from an internal validation subset could improve estimates and adjust precision for random error in prevalence, sensitivity, and specificity, while still providing substantial overall cost-savings compared to individual PCR testing.

Overall, one-step pooled qPCR testing that assumed perfect sensitivity and specificity underestimated the prevalence of malaria parasitaemia among RDT negative samples compared to the gold standard of individual qPCR testing. This estimate substantially improved after adjustment for sensitivity and specificity, highlighting the importance of including a validation subset for studies that use pooled testing. The use of validation samples to adjust for misclassification of binary outcomes has been well described [30]. However, validation data derived from external data sources may provide biased information if the validation population is not representative of the main study population (issue of “transportability”) [28]. Additionally, mis-substitution bias can occur in the absence of a gold standard to obtain valid estimates of sensitivity and specificity [31]. In the present study, prevalence estimates by pooled testing were less subject to these biases due to the availability of a gold standard (individual qPCR testing) and fixed pool sizes. The value of combining both validation and main study data [28] from the same study population was illustrated and provided a more accurate prevalence estimation while appropriately accounting for the uncertainty in detection due to imperfect sensitivity and specificity reflected in the validation subset when quantifying the precision of the prevalence estimate.

In low prevalence contexts (< 10%), pooled testing can be more precise compared to individual testing [25]. However, the prevalence of subpatent parasitaemia in the overall study area is slightly above this threshold and an increase in variance with transmission setting was observed. Tu et al. showed that as the probability of having more than one positive sample in the pool increases, the precision of pooled testing declines compared to individual testing [25]. In this study, given the higher probability of multiple positive samples in a pool, pooled testing did not offer a precision advantage over individual sample testing, but the wider confidence intervals were still within an acceptable range after adjusting for imperfect sensitivity and specificity. This again highlights the utility of including a validation subset derived from the same study population as the main samples for estimates using one-step pooled testing.

Given that qPCR provides quantification of malaria parasites, in addition to a binary positive or negative qPCR result, continuous measures of parasite density of individual samples and pools were determined. The results showed that the GMPD of positive individual samples was significantly higher in pools that tested positive (true positive pools) compared to pools that tested negative (false negative pools). This suggests that the sensitivity of a pooled testing strategy may depend on not only the number of positive samples in the pool, but also the parasite density of the samples. Other studies have reported dilution effects of pooling, resulting in loss of sensitivity as pool sizes become larger [7, 32]. This finding may have implications in the design of pooling assays and selection of pool sizes in lower transmission settings where a majority of infections harbor lower parasite densities.

This study also observed an inverse relationship between the sensitivity of one-step pooling in RDT negatives and transmission intensity. Although sensitivity and specificity are characteristics of a test and believed to be independent of underlying disease prevalence, there are certain mechanisms that may result in a dependence between test characteristics and prevalence [33]. Such dependence could occur if pathogen load is affected by the prevalence of disease, leading to differences in the proportion of infections harboring densities below the limit of detection of the diagnostic test [34, 35]. This dependence may explain the lower sensitivity of one-step pooling in Karemo compared to Asembo.

The one-step pooled qPCR testing strategy provided considerable consumable cost savings compared to individual qPCR testing. The additional inclusion of a validation subset, in which ~ 20% of samples would be run individually, still offered a cost-saving alternative (52% reduction) to individual sample testing. Other malaria studies that have applied pooled strategies for other outcomes have estimated cost reductions similar to this study (> 50%) [14, 19]. The present study also reported on the time saving potential of pooled strategies from the perspective of laboratory staff hands-on time. Although this estimate is somewhat subjective and would not be comparable with robotic high-throughput automation, the inclusion of a time-saving estimate provides information for the feasibility of pooled strategies as a resource saving alternative to obtain molecular results.

There were several considerations related to the sampling approach that should be weighed in future studies. First, since this study included a random sample of RDT negative participants rather than all participants, it was not possible to estimate the population prevalence of low parasite density infections. Low density infections at the tail end of parasite clearance (with persistent antigenemia [36]) or those harboring histidine-rich protein 2/3 gene deletions (not detected currently in our study areas in western Kenya, unpublished data) may have been among RDT positive cases not sampled in this study. However, chronic low density infections (rather than those that arise towards the end of an infection), which were more likely to be detected in this study, may be more important for onward transmission [5]. Second, a random sample was taken across all age groups. Given that the development of partial malaria immunity increases with age, adults are more likely to harbor low density infections [1], and oversampling of certain age groups and/or a stratified pooling approach may be necessary to address certain questions.

Finally, this study included a ~ 20% sample for the validation subset [26–28]; however, additional studies could consider design-based approaches to calculate an optimal sample size for the validation subset with consideration of underlying disease prevalence and desired precision.

## Conclusion

This study illustrated a novel application of MLE analytic procedures to estimate the prevalence of malaria parasitaemia among RDT negative samples by a one-step pooled molecular testing strategy. The results demonstrated that the accuracy of estimates could be restored after adjustment for misclassification in pooled assays using the sensitivity and specificity determined in an internal validation subset derived from the study population and, additionally, that uncertainty in those estimates can be accounted for by applying an inverse-variance weighting approach. Although the outcome prevalence was just above the optimal range for pooling, with the inclusion of validation data derived from the same study population, pooled testing provided accurate prevalence estimates without materially compromising precision. One-step pooled testing designs with validation data should be considered as a cost-saving alternative to expand the utility of molecular testing for surveillance of low prevalence outcomes.

## Supplementary Information

**Additional file 1.** Methods for sample size determination and sampling weights.

## Data Availability

The dataset used in this study is available and can be shared upon reasonable request to the corresponding authors.
