# Supplementary material for: Novel application of one-step pooled molecular testing and maximum likelihood approaches to estimate the prevalence of malaria parasitaemia among rapid diagnostic test negative samples in western Kenya
Source: Malar J. 2022 Nov 6;21:319. doi: 10.1186/s12936-022-04323-2 (PMC9638440; doi:10.1186/s12936-022-04323-2)
Supplement: Supplementary file 1 — Additional file 1. Methods for sample size determination and sampling weights. [file 12936_2022_4323_MOESM1_ESM.docx]

# Additional Methods

## Pilot study used to determine sample size (cited in the subsection of sample size and selection)

A pilot study in a random sample of 107 rapid diagnostic test (RDT) negative round one participants was conducted to determine the variability of the quantitative PCR (qPCR) assay in this population. Based on these results and to obtain stratified estimates by transmission setting (Asembo [lowest, relative to study area], Gem [middle], and Karemo [highest]), it was determined that using a pool size of 5 samples with a desired precision of +/- 2-4% and expected number of positives per pool <1.6 [24] required a minimum number of 860 pools in total or 335 pools (1675 samples) from Asembo, 390 pools (1950 samples) from Gem, and 135 pools (675 samples) from Karemo. The sample size was increased by approximately 40 additional pools (200 samples) per area to account for potential exclusion of poor-quality samples or for sample unavailability.

## Sampling weights (cited in the subsection of data and statistical analyses)

Sampling weights were applied for overall prevalence estimates. Weights were defined as the number of RDT negative participants divided by the total number of RDT negative participants in all three areas combined. The number of RDT negative participants in Asembo, Gem, and Karemo, respectively, were 3,207, 6,680 and 3,552, corresponding to weights of 0.239, 0.497, and 0.264.

Each area-specific weight was multiplied by the area-specific prevalence estimate and the sum of this product provided the overall prevalence estimate. The variance of the overall prevalence estimate was calculated as the sum of the area-specific weight squared multiplied by the area-specific variance.
